# Development of anti-glomerular basement membrane glomerulonephritis during the course of IgA nephropathy: a case report

**DOI:** 10.1186/s12882-019-1207-3

**Published:** 2019-01-25

**Authors:** Tadasu Kojima, Go Hirose, Shuuhei Komatsu, Taito Oshima, Kentaro Sugisaki, Tomohiro Tomiyasu, Noriko Yoshikawa, Muneharu Yamada, Takashi Oda

**Affiliations:** Department of Nephrology and Blood Purification, Kidney Disease Center, Tokyo Medical University Hachioji Medical Center, 1163 Tatemachi, Hachioji, Tokyo 193-0998 Japan

**Keywords:** Anti-glomerular basement membrane (GBM) glomerulonephritis, Rapidly progressive glomerulonephritis (RPGN), IgA nephropathy, Crescentic glomerulonephritis

## Abstract

**Background:**

Anti-glomerular basement membrane (GBM) glomerulonephritis does not usually coexist with another glomerulonephritis such as IgA nephropathy. We present a rare case having a combination of these two diseases, and furthermore, histological evaluation could be performed before and after the development of anti-GBM glomerulonephritis over a period of only10 months.

**Case presentation:**

A 66-year-old woman was admitted with complaints of microscopic hematuria and mild proteinuria for the past 3 years. Serum creatinine level was normal at that time. The first renal biopsy was performed. Light microscopy revealed mesangial proliferative glomerulonephritis with fibro-cellular crescents in one out of 18 glomeruli, excluding one global sclerotic glomerulus. Immunofluorescence (IF) showed IgA and C3 deposition in the mesangium. Therefore, the diagnosis was IgA nephropathy. Eight months later, the patient’s serum creatinine suddenly rose to 4.53 mg/dL and urinalysis showed 100 red blood cells per high power field with nephrotic range proteinuria (12.3 g/g_Cr_). The serological tests revealed the presence of anti-GBM antibody at the titer of 116 IU/mL. Treatments were begun after admission, consisting of hemodialysis, plasma exchange, and intravenous methylprednisolone pulse therapy. At 4 weeks after admission, the second renal biopsy was performed. Light microscopy revealed crescents in 18 of 25 glomeruli, excluding six global sclerotic glomeruli. IF showed linear IgG deposition along the GBM in addition to granular IgA and C3 deposition. Based on these findings, the diagnosis of anti-GBM glomerulonephritis and IgA nephropathy was confirmed. Renal function was not restored despite treatment, but alveolar hemorrhage was prevented.

**Conclusions:**

We report a patient with a diagnosis of anti-GBM disease during the course of IgA nephropathy. This case strongly suggests that the presence of autoantibodies should be checked to rule out overlapping autoimmune conditions even in patient who have previously been diagnosed with chronic glomerulonephritis, such as IgA nephropathy, who present an unusually rapid clinical course.

## Background

Anti-glomerular basement membrane (GBM) glomerulonephritis is an autoimmune glomerular disease that is characterized by linear deposition of IgG along the GBM. The main target of anti-GBM antibody had been shown to lie in the NC1 domain of α3 chains of type IV collagen on the GBM. Histologically, it is associated with extensive crescent formation and clinically with rapidly progressive glomerulonephritis (RPGN). There are well-known associations between anti-GBM glomerulonephritis and the presence of antineutrophil cytoplasmic antibody (ANCA) and between anti-GBM glomerulonephritis and membranous nephropathy [1–3]. In a limited number of cases, anti-GBM glomerulonephritis has been associated with IgA nephropathy and other immune complex glomerulonephritis [4–6].

Herein, we report a patient with a diagnosis of anti-GBM disease during the course of IgA nephropathy.

## Case presentation

**Fig. 1:**
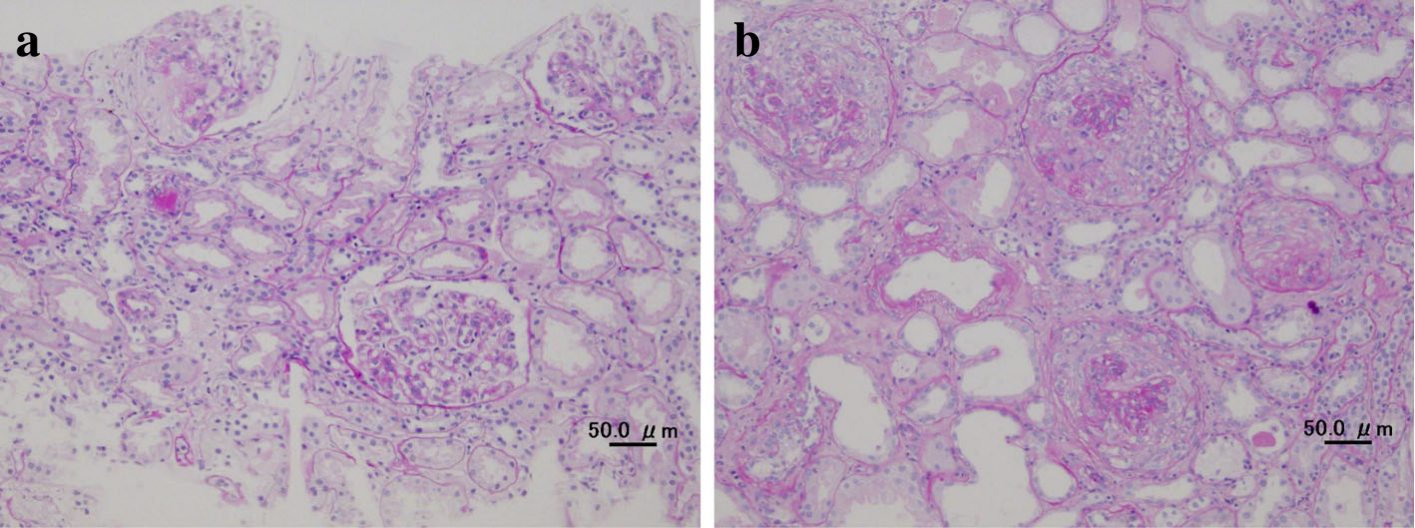
Representative photographs of Periodic acid-Schiff stained sections (Scale bars = 50.0 μm). **a** First renal biopsy showing mesangial proliferative glomerulonephritis with fibro-cellular crescent in one glomerulus. **b** Second renal biopsy showing diffuse crescent formation and a few residual glomerular tufts. Scale bars = 50.0 μm

**Fig. 2:**
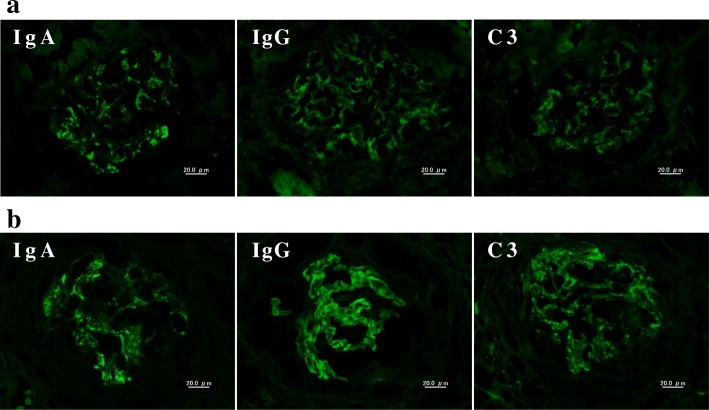
Representative photographs of immunofluorescence staining (Scale bars = 20.0 μm). **a** First renal biopsy showing positive staining of IgA, IgG, and C3. The staining pattern was similar in IgA and C3 (granular deposition probably in the mesangial area), but was rather different in IgG. **b** Second renal biopsy showing linear immunofluorescence for IgG along the glomerular capillary walls. IgA staining was found in mesangial areas, whereas C3 deposition was observed in mesangial areas as well as partially in the glomerular capillary walls

**Fig. 3:**
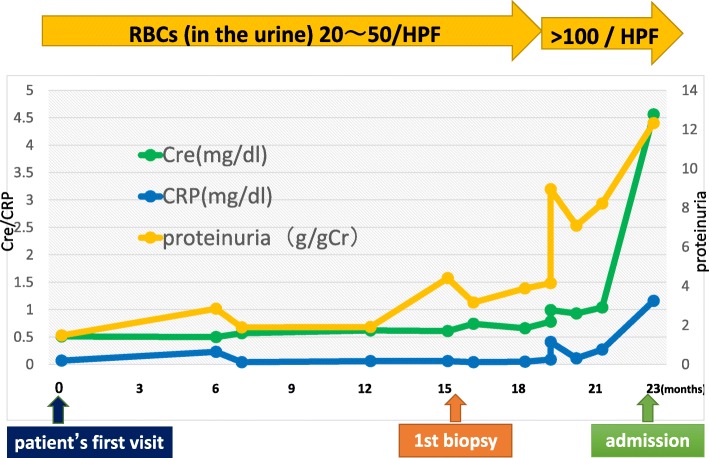
Clinical course before admission. Cre: serum creatinine level

A 66-year-old woman with a significant past medical history of well-controlled hypertension was admitted with complaints of microscopic hematuria and mild proteinuria for the past 3 years. Serum creatinine level was within normal range at that time and therefore the anti-GBM antibody was not tested. The first renal biopsy revealed mesangial proliferative glomerulonephritis with fibro-cellular crescents in one out of 18 glomeruli, excluding one global sclerotic glomerulus (Fig. 1a), and deposition of IgA and C3 in mesangial areas by immunofluorescence microscopy (Fig. 2a). Weak but significant IgG deposition was also observed in glomeruli in the distribution somewhat different from IgA or C3 (Fig. 2a). The electron-dense deposits were observed in mesangial areas by electron microscopy. Therefore, the diagnosis was IgA nephropathy. Antihypertensive therapy was initiated, mainly with an RAS inhibitor. Eight months later, the patient’s serum creatinine suddenly rose to 4.53 mg/dL (it was 1.04 mg/dL from the routine blood test 1 month before). Urinalysis showed 100 red blood cells per high power field and urinary protein excretion of 12.3 g/g_Cr_ (Fig. 3). The serological tests that were performed to differentiate the cause of rapidly progressive glomerulonephritis revealed the presence of anti-GBM antibody at the titer of 116 IU/mL and the absence of anti-nuclear antibody and anti-neutrophil cytoplasmic antibody. Laboratory findings on admission are summarized in the Table 1.

**Table 1:** Laboratory data on admission

CBC	
WBC (4000–8000)	10,500/μL
RBC (390–510)	379 × 10^4^/μL
Hb (12.0–16.0)	11.3 g/dL
PLT (15–35)	36.9 × 10^4^/μL
Chemistry	
TP (6.4–8.0)	6.4 g/dL
Alb (3.4–5.0)	2.8 g/dL
AST (8–40)	30 U/L
ALT (4–43)	21 U/L
LDH (106–220)	345 mg/dL
BUN (8–20)	35.6 mg/dL
Cr (0.5–0.8)	5.53 mg/dL
Na (135–147)	136 mEq/L
K (3.4–4.9)	3.9 mEq/L
Cl (98–108)	103 mEq/L
Ca (8.0–10.5)	7.9 mg/dL
P (2.7–4.5)	6.1 mg/dL
CRP (< 0.02)	1.16 mg/dL
Immune-related	
C3 (86–160)	145 mg/dL
C4 (17–45)	51.3 g/dL
CH50 (30–45)	76.8 U/mL
IgG (380–1620)	881 mg/dL
IgA (84–438)	186 mg/dL
IgM (57–288)	33 mg/dL
ASO (< 166)	22 IU/mL
ANA (< 40)	< 40
Anti –GBM antibody (< 3.0)	116 IU/mL
PR3-ANCA (< 3.5)	<1.0 IU/mL
MPO-ANCA (< 3.5)	<1.0 IU/mL
Urinalysis	
Occult blood	3+
RBCs	>100/HPF
Protein	4 + 12.3 g/gCr
WBC	1–4/HPF
Cast	Granular cast(+)
	RBCcast(+)

*WBC* white blood cells, *RBC* red blood cells, *Hb* hemoglobin, *Plt* platelets, *HPF* high-power field, *TP* total protein, *Alb* albumin, *AST* aspartate aminotransferase, *ALT* alanine aminotransferase, *LDH* lactate dehydrogenase, *BUN* blood urea nitrogen, *Cr* creatinine, *Na* sodium, *K* potassium, *Cl* chloride, *Ca* calcium, *P* phosphate, *CRP* C-reactive protein, *ANA* anti-nuclear antibody, *GBM* glomerular basement membrane, *ANCA* anti-neutrophil cytoplasmic antibody, *PR3* proteinase 3, *MPO* myeloperoxidase

**Fig. 4:**
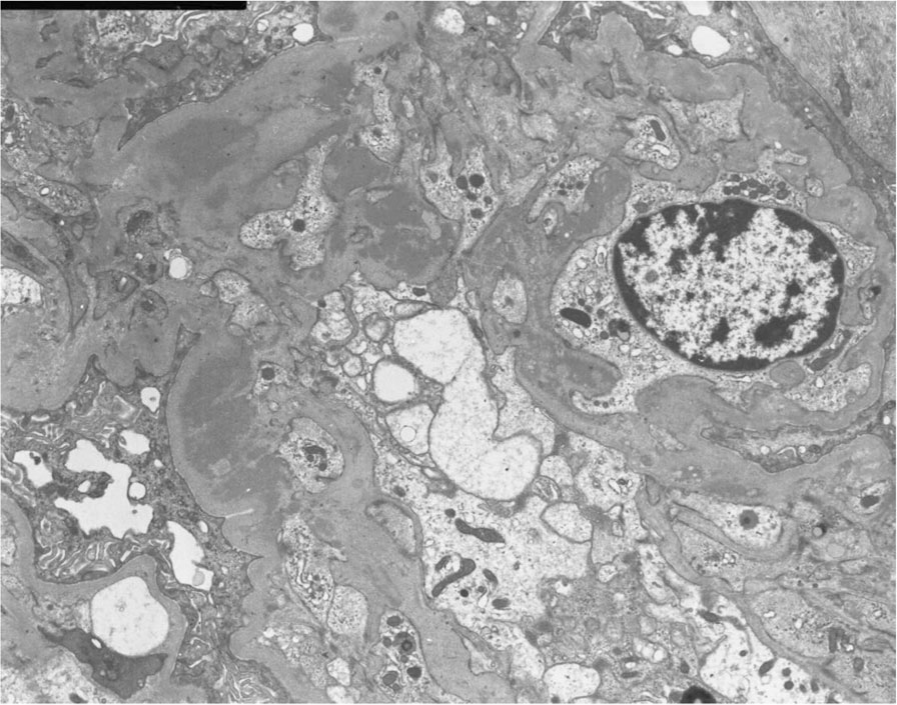
Electron microscopic photograph of the second renal biopsy, showing the electron-dense deposits in mesangial areas

**Fig. 5:**
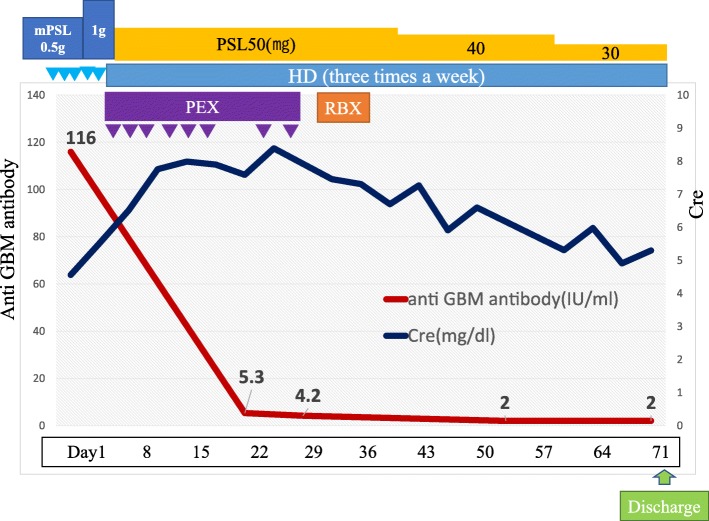
Clinical course after admission. Cre: serum creatinine level, Anti-GBM antibody: anti-glomerular basement membrane antibody, PEX: plasma exchange, mPSL: methylprednisolone, PSL: prednisolone, HD: hemodialysis, RBX: renal biopsy

After admission, treatments with hemodialysis, plasma exchange, and intravenous methylprednisolone pulse therapy followed by oral prednisolone at the dose of 50 mg/day were initiated. The second renal biopsy was performed at 4 weeks after admission in order to assess the probability of renal recovery and to make the final diagnosis. It revealed cellular to fibrocellular crescents in 18 of 25 glomeruli, excluding six global sclerotic glomeruli by light microscopy. By immunofluorescence study, linear IgG deposition along the glomerular capillary walls and mesangial staining for IgA were observed. On the other hand, C3 deposition was observed in the mesangium as well as in the glomerular capillary walls (Fig. 2b). Electron-dense deposits were observed in mesangial areas, similarly as in the first biopsy, by electron microscopy (Fig. 4). Based on the aforementioned findings, the diagnosis of anti-GBM glomerulonephritis and IgA nephropathy was confirmed. Plasmapheresis was performed eight times, anti-GBM antibody gradually decreased, and alveolar hemorrhage was prevented. However, her renal function could not be restored and she underwent maintenance hemodialysis (Fig. 5).

Additional immunosuppressant was not given because the patient did not show any sign of pulmonary involvement and because the renal recovery was quite unlikely from clinical (continuous oliguria and hemodialysis dependence) as well as histological (crescent formation in most of non-sclerotic glomeruli) point of view.

Clinical and histological presentations from IgA nephropathy (at the time of first renal biopsy) and from anti-GBM disease (at the time of second renal biopsy) were summarized in the Table 2.

**Table 2:** Clinical and histological presentation at the time of first and second renal biopsy

	First biopsy	Second biopsy
Clinical presentation	CGN	RPGN
	U-P 3.2 g/g·Cr	U-P 12.3 g/g·Cr
	U-RBC 50–99/HPF	U-RBC > 100/HPF
	sCr 0.74 mg/dL	sCr 5.5 mg/dL
Anti-GBM antibody	Not tested	116 IU/ml
Light microscopy	Mesangial proliferative GN	Crescentic GN
	Global sclerosis (1/19)	Global sclerosis (6/31)
	Fibrocellular crescent (1/19)	Cellular~fibrocellular crecent (18/31)
Immunofluorescence	IgA: mes ++	IgA: mes +
	C3: mes ++	C3: mes +
	IgG: mes +,	peripheral linear +
	peripheral linear ±	IgG: peripheral linear ++
	(focal segmental)	(diffuse global)

*CGN* chronic glomerulonephritis, *RPGN* rapidly progressive glomerulonephritis, *GBM* glomerular basement membrane, *GN* glomerulonephritis, *mes* mesangium

## Discussion and conclusions

IgA nephropathy is an immune complex-mediated glomerulonephritis defined immunohistologically by the presence of glomerular mesangial IgA deposits accompanied by a variety of histopathologic lesions, including mesangial proliferation [7]. Anti-GBM disease is caused by antibodies reactive to the glomerular and alveolar basement membrane.

**Fig. 6:**
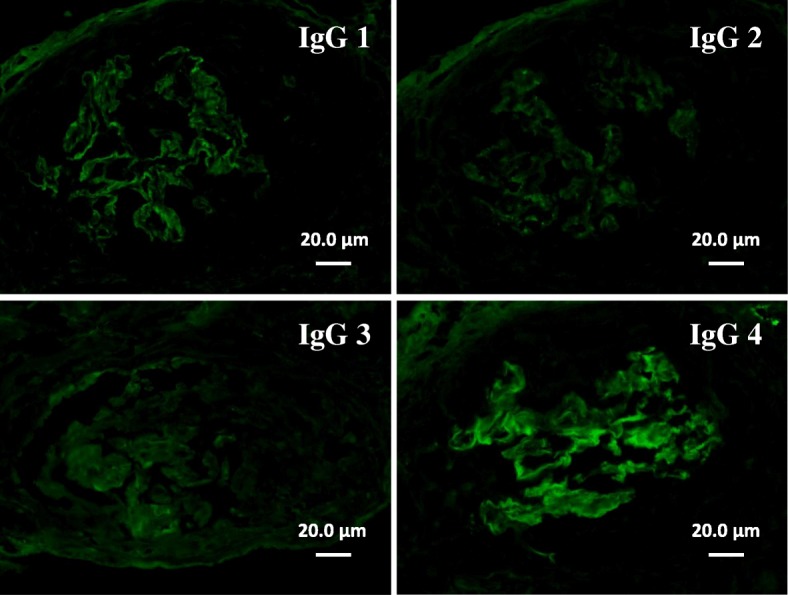
Immunofluorescence staining for IgG subclasses on the second renal biopsy (Scale bars = 20.0 μm). The main subclass of IgG bound to GBM was IgG4

The causal relationship of anti-GBM glomerulonephritis and IgA nephropathy is unclear. There was one hypothesis that the IgA-related immune complex might promote immunologic and inflammatory events, resulting in conformational changes and exposure of the GBM antigens leading to development of anti-GBM antibody [4]. However, it is difficult to prove whether anti-GBM disease in this patient developed as an incidental complication or was secondary to IgA nephropathy because there is still no established marker to distinguish primary from secondary anti-GBM disease. In this regard, we performed immunofluorescence staining for IgG subclasses on the second renal biopsy, and found that IgG4 was the main subclass of IgG bound to GBM in this patient (Fig. 6). The main subclass of pathogenic IgG in anti-GBM disease was reported to be usually IgG1 [8]. Whether predominance of IgG4 relates with anti-GBM disease developed secondary to IgA nephropathy deserves future study.

**Fig. 7:**
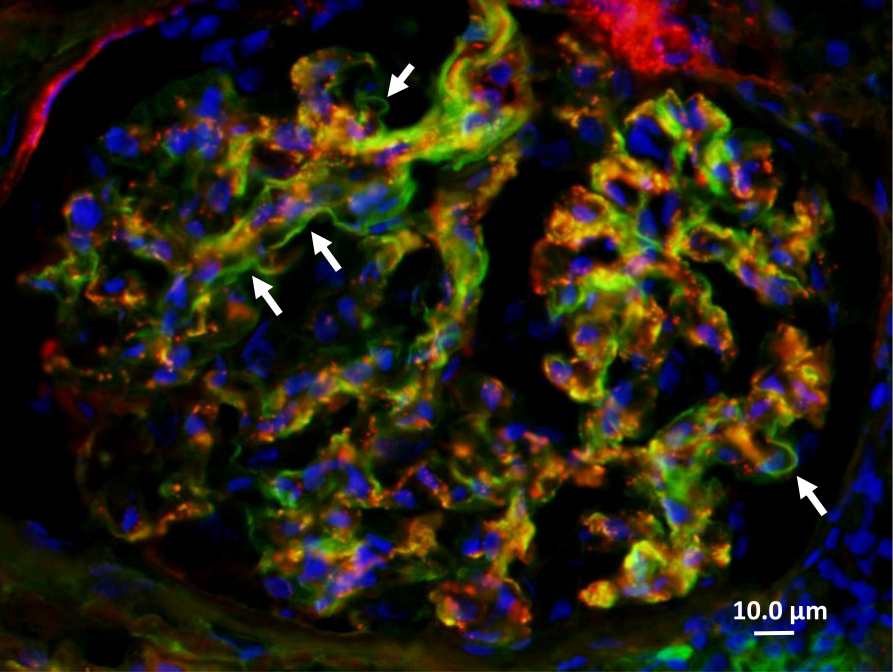
Multiple immunofluorescence staining for IgG (FITC: green), C3 (Alexa Fluor 594: red) and DAPI (blue) on the first biopsy. A linear IgG-positive portion was observed partially along the glomerular capillary walls as indicated by white arrows. Scale bar = 10.0 μm

The pathophysiological condition of anti-GBM disease before clinical presentation is unknown. In this regard, Olson et al. conducted a case-control study involving 30 patients diagnosed with anti-GBM disease using serum samples from the Department of Defense Serum Repository. In the report, 13% (4 of 30) of study subjects had an elevated anti-GBM antibody level 2–10 months prior to diagnosis [9]. In the present case, multiple immunofluorescence labelling on the first biopsy showed partial linear IgG deposition along the glomerular capillary walls (Fig. 7). Although it may be only speculation because serum anti-GBM antibody was not tested at the time of the first biopsy, the patient might have had complications from asymptomatic (subclinical) anti-GBM disease at that point.

In the case of IgA nephropathy complicated by anti-GBM disease, Yamaguchi et al. speculated that the pathological features of IgA nephropathy may not be observed because the number of glomeruli free from destruction is very limited [10]. Therefore, the coexistence of anti-GBM glomerulonephritis and IgA nephropathy may be more frequent than is being reported.

In summary, we reported a patient with a diagnosis of anti-GBM disease during the course of IgA nephropathy. Histological evaluation could be performed before and after the development of anti-GBM disease over a period of 10 months. Even if the patient had already received a diagnosis of a chronic glomerulonephritis such as IgA nephropathy, we should check autoantibodies to rule out overlapping autoimmune conditions in case the patient showed an unusually rapid clinical course.

## Abbreviations

ANCA: Antineutrophil cytoplasmic antibody
GBM: Glomerular basement membrane
IF: Immunofluorescence
RPGN: Rapidly progressive glomerulonephritis
